# Supplementary material for: Doxorubicin-Loaded Core–Shell UiO-66@SiO2 Metal–Organic Frameworks for Targeted Cellular Uptake and Cancer Treatment
Source: Pharmaceutics. 2022 Jun 23;14(7):1325. doi: 10.3390/pharmaceutics14071325 (PMC9324125; doi:10.3390/pharmaceutics14071325)
Supplement: Supplementary file 1 [file pharmaceutics-14-01325-s001.zip › pharmaceutics-1734284-supplementary.pdf]

Supporting information

for

Doxorubicin-loaded core-shell UiO-66@SiO_2 metal-organic frameworks for targeted cellular uptake and cancer treatment

Daria B. Trushina[1,2], Anastasia Yu. Sapach[2,3], Olga Burachevskaia[4], Pavel V. Medvedev[4], Dmitry N. Khmelenin[1], Tatiana N. Borodina[1], Mikhail A. Soldatov[4], Vera V. Butova[4*]

[1] Federal Research Center Crystallography and Photonics, Russian Academy of Sciences, Moscow 119991 Russia

[2] Sechenov First State Medical University, Moscow 119991 Russia

[3] Skolkovo Institute of Science and Technology, Moscow 143025, Russia

[4] The Smart Materials Research Institute, Southern Federal University, Rostov-on-Don 344090, Russia

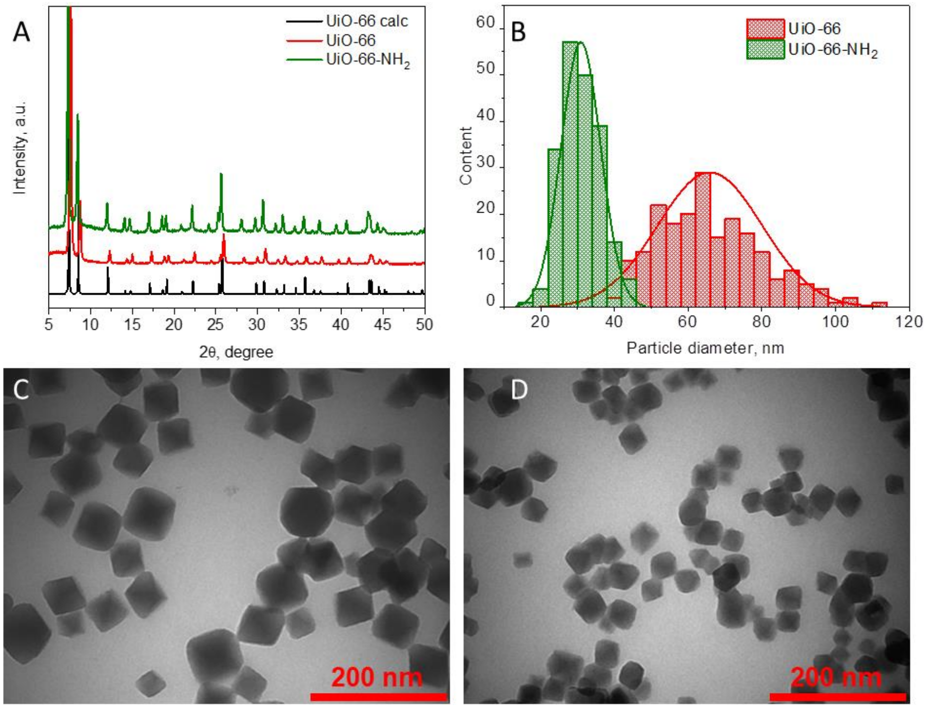

Figure S1. Powder XRD patterns of samples UiO-66 and UiO-66 NH2 (A). Profile UiO-66 calc was calculated according to crystallographic information from 10.1021/cm1022882. Particle-sized distribution according to TEM images from 200 particles (B). Representative TEM images of UiO-66 (C) and UiO-66-NH_2 (D) samples.

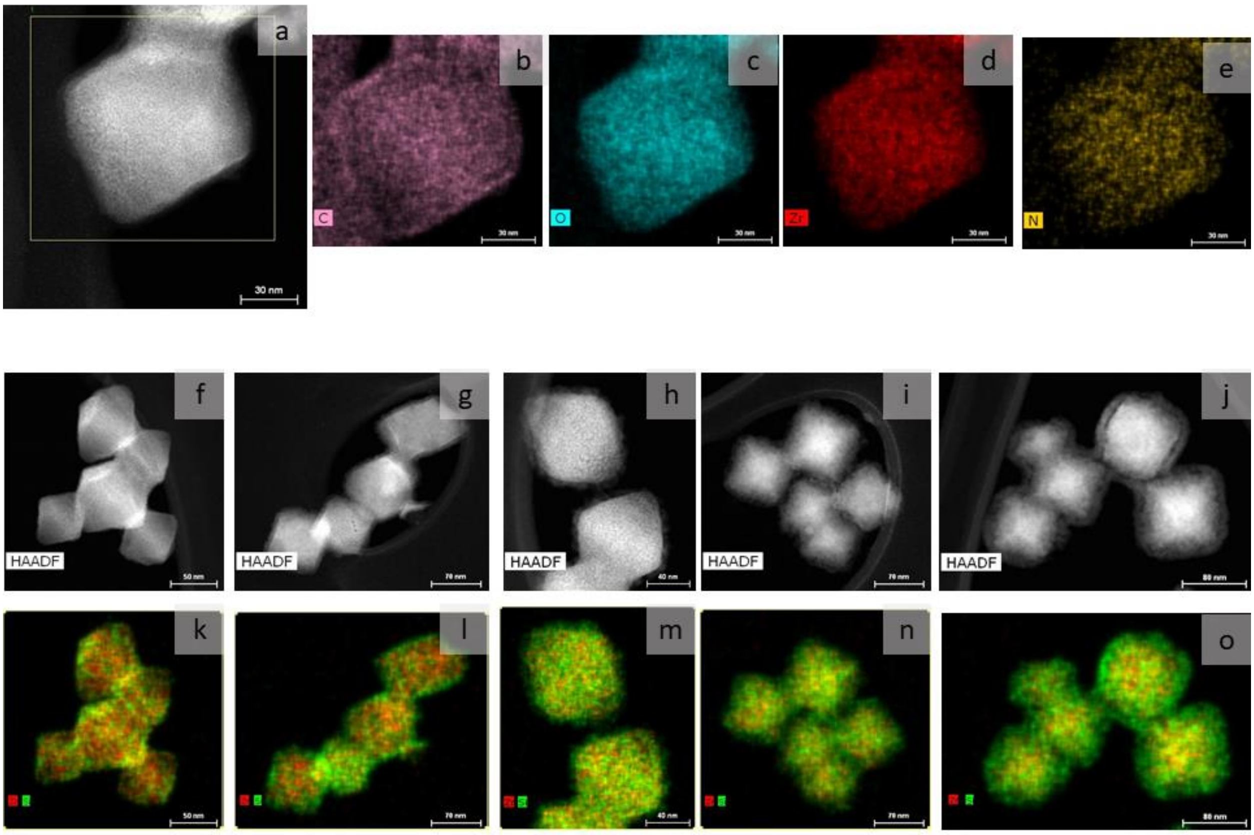

Figure S2. TEM images of UiO-66-NH_2 MOFs with maps of C, O, Zr, and N (a-e). HAADF images and corresponding Zr-Si elemental maps for UiO-66-NH_2@SiO2 samples obtained by incubation of nanoparticles in TEOS for 0.5h (f, k), 1h (g, l), 2h (h, m), 4h (i, n), 24h (j, o).

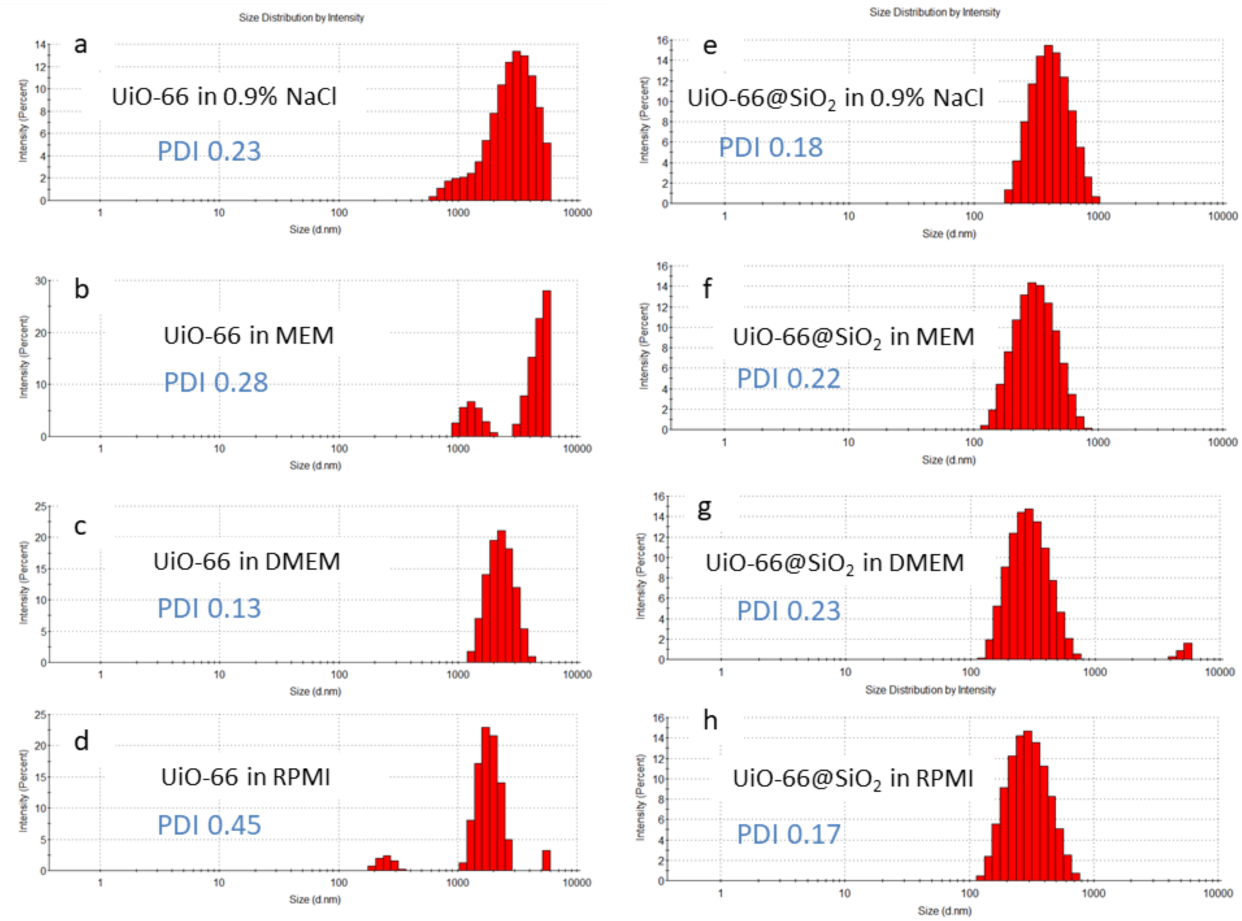

Figure S3. DLS-intensity results for UIO-66 and UiO-66@SiO_2 MOFs dispersed in saline solution, MEM, DMEM and RPMI cell culture media.

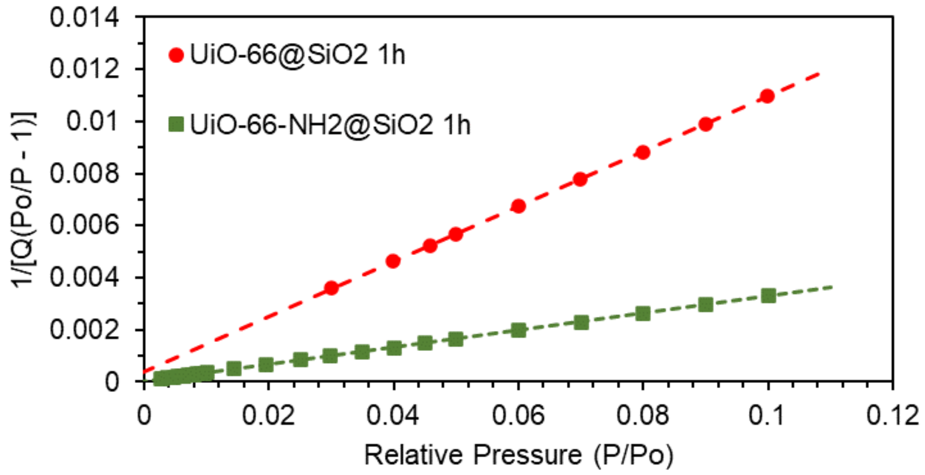

Figure S4. **B**ET surface area plots for UiO-66@SiO_2 1h (red) and UiO-66-NH_2@SiO_2 1h (green) samples. Markers represent experimental data; dashed lines show BET approximation.

Table S1. Details of porosity calculations. SSA stands for specific surface area, C is the BET constant.

	BET			Total pore volume, mm^3/g	t-plot calculations			
	SSA, m^2/g	C	Correlation coefficient		Thickness equation	Micropore volume, mm^3/g	Micropore area, m^2/g	Correlation coefficient
UiO-66@SiO_2 1h	40.0	457.2	0.99969	55.466	Harkins and Jura	8.996	22.1	0.993088
UiO-66-NH_2@SiO_2 1h	133.3	1414.4	0.99996	99.455		38.95	98.3	0.996116

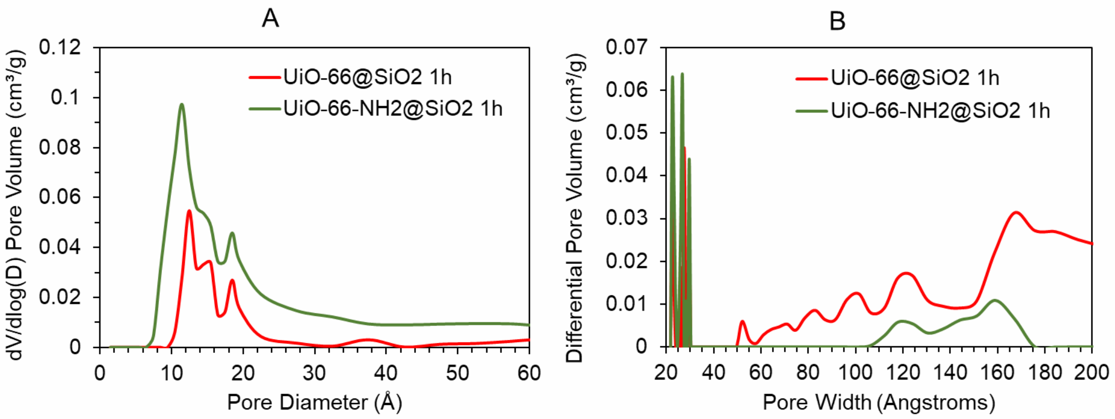

Figure S5. (A) BJH pore size distribution according to adsorption branches of isotherms. (B) Pore size distribution by NLDFT according to the model of cylindrical pores in an oxide surface.

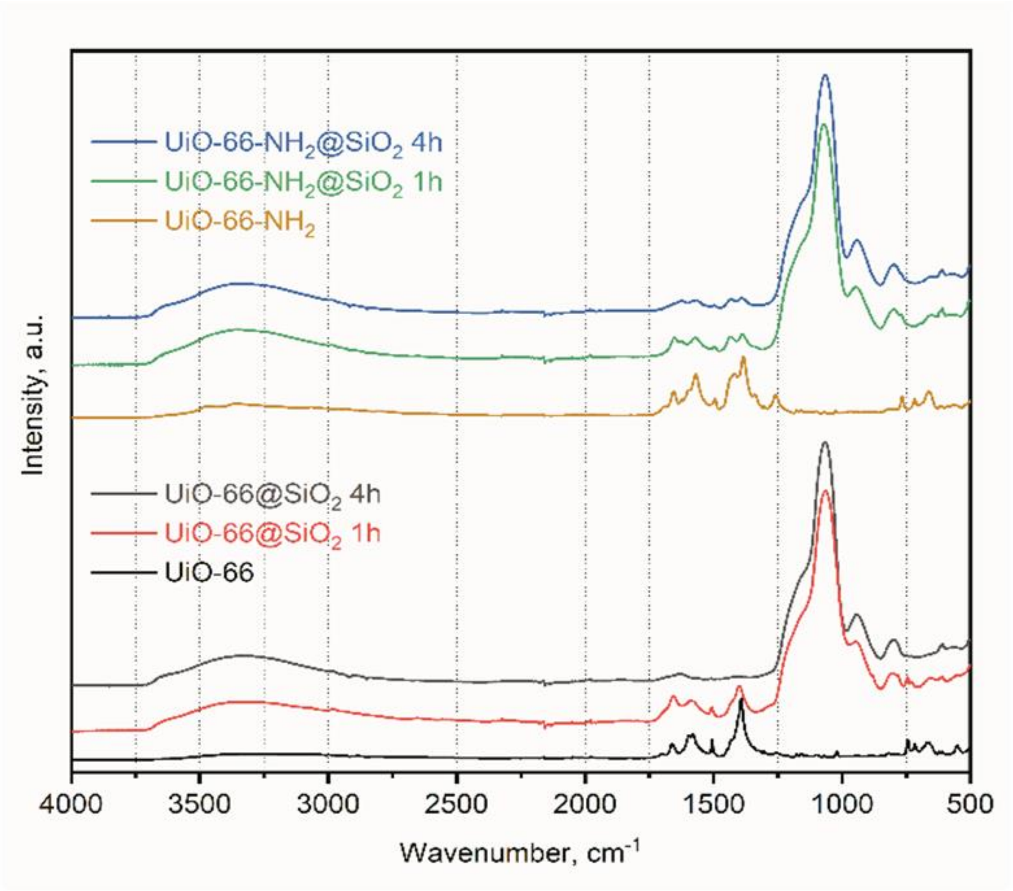

Figure S6. ATR-FTIR spectra collected for intact UiO-66 and UiO-66-NH_2 nanoparticles compared to UiO-66@SiO_2 and UiO-66-NH_2@SiO_2 MOFs treated with TEOS for 1 and 4 h.

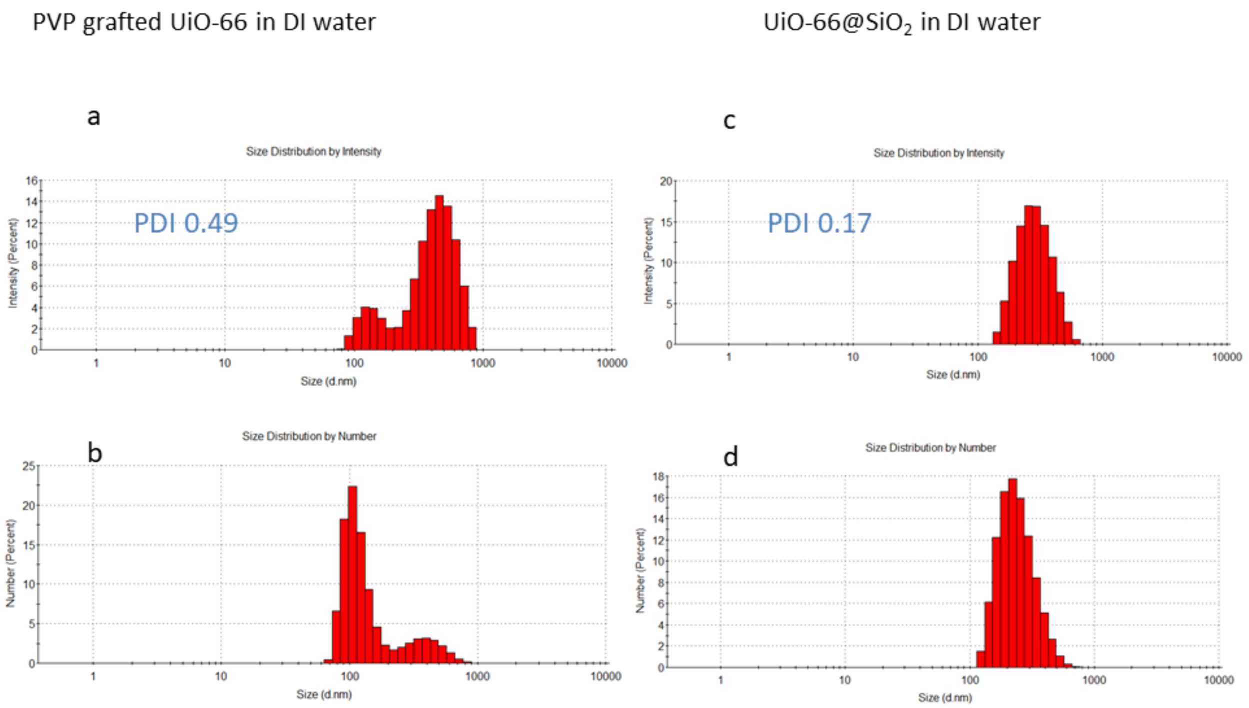

Figure S7. DLS-intensity and DLS-number results for PVP grafted UiO-66 and UiO-66@SiO_2 water dispersions.

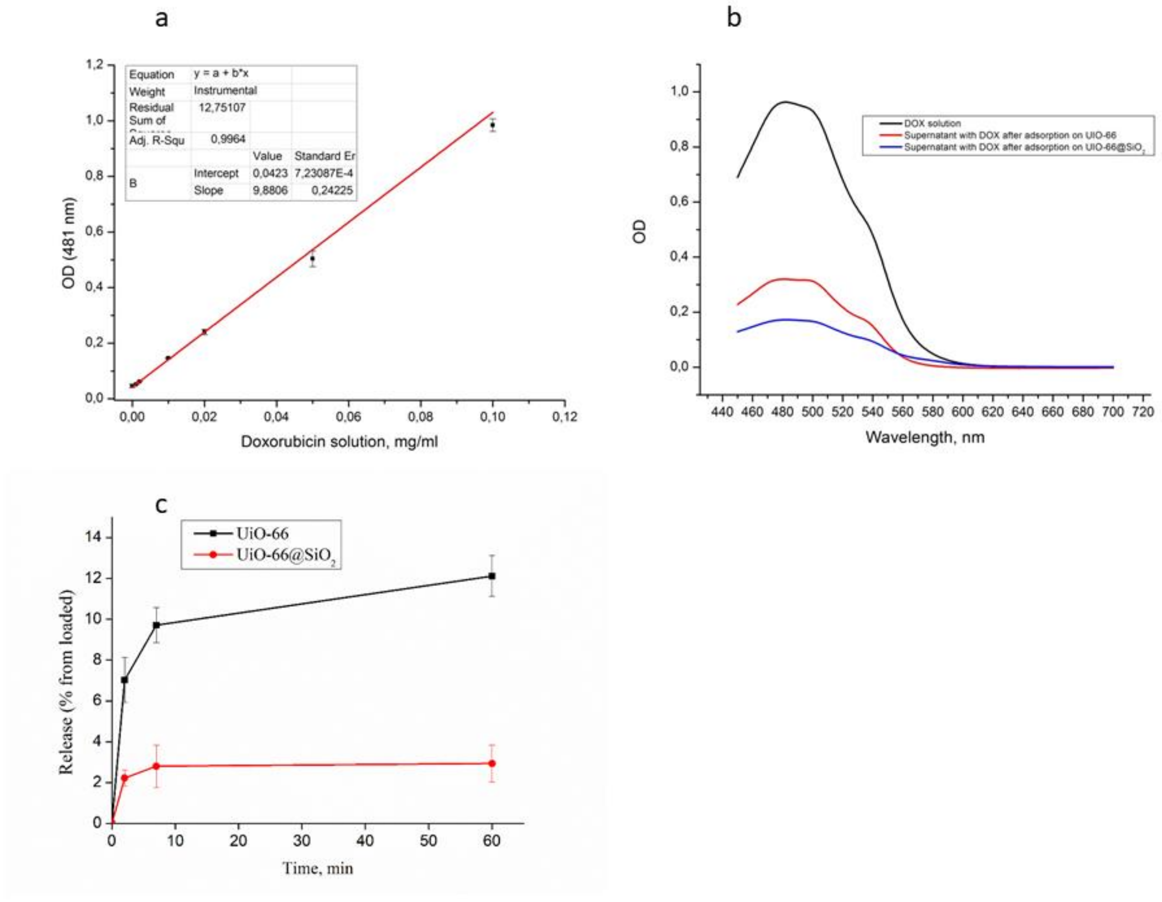

Figure S8. Calibration curve for DOX (a) and absorption spectrum of the DOX solution and DOX in supernatants after loading into MOFs (b), DOX release profiles from UiO-66 and UiO-66@SiO_2 (c) .

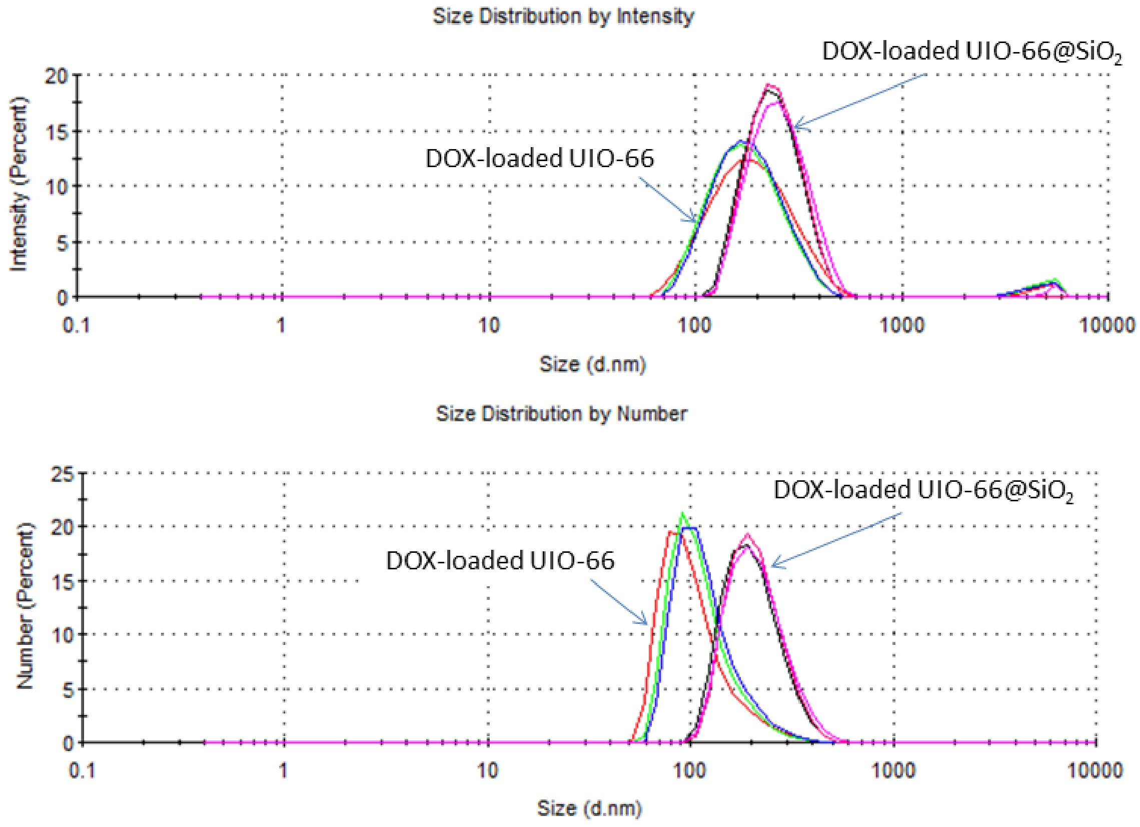

Figure S9. DLS-intensity and DLS-number distributions DOX-loaded UIO-66 and UIO-66@SiO_2.

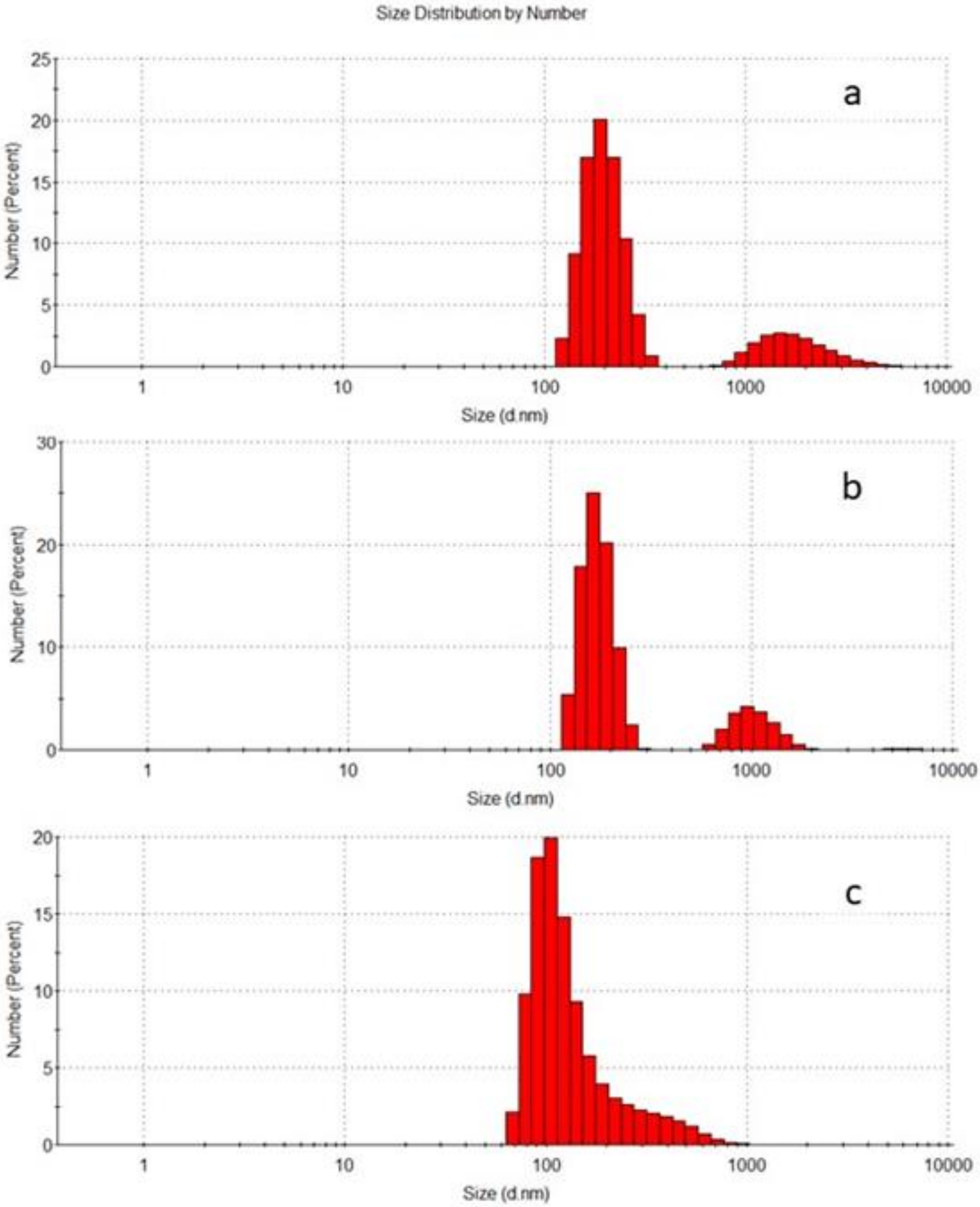

Figure S10. DLS-number distributions of DOX-loaded UiO-66/F127-FA in MEM (a), DMEM (b), RPMI (c) cultural media.
